# Primary gastric squamous cell carcinoma: case report

**DOI:** 10.1093/jscr/rjad736

**Published:** 2024-01-16

**Authors:** Vasco S Cardoso, Filipa C Costa, Cristina M Santos, José M Guerreiro, Sância F Ramos

**Affiliations:** General Surgery Department – Centro Hospitalar Lisboa Ocidental, São Francisco Xavier’s Hospital, 1449-005 Lisboa, Portugal; General Surgery Department – Centro Hospitalar Lisboa Ocidental, São Francisco Xavier’s Hospital, 1449-005 Lisboa, Portugal; General Surgery Department – Centro Hospitalar Lisboa Ocidental, São Francisco Xavier’s Hospital, 1449-005 Lisboa, Portugal; General Surgery Department – Centro Hospitalar Lisboa Ocidental, São Francisco Xavier’s Hospital, 1449-005 Lisboa, Portugal; Anatomopathological Department – Centro Hospitalar Lisboa Ocidental, São Francisco Xavier’s Hospital, 1449-005 Lisboa, Portugal

**Keywords:** gastric cancer, primary gastric squamous cell carcinoma, gastric surgery, gastric cancer treatment

## Abstract

Primary gastric squamous cell carcinoma is an extremely rare entity with an unknown etiopathology, and prognosis is generally poor. While diagnostic criteria are standardized, the optimal treatment strategy is not defined due to its rarity. In this article, we present a case of primary gastric squamous cell carcinoma, discussing its approach and treatment.

## Introduction

Primary gastric squamous cell carcinoma (PGSCC), first described in 1905, is a rare entity with fewer than 100 cases reported in the literature [1, 2]. It represents 0.04–0.07% of all gastric cancers, with an incidence ratio of 5:1 in men to women [1–3]. It is more common in the sixth decade of life, with most cases described in the upper third of the stomach [2]. The exact pathogenesis remains unclear, and the optimal treatment strategy is yet to be defined [2, 3].

## Case report

A 66-year-old female patient was referred to our department with complaints of nausea, early satiety and epigastric pain over the last 2 months. She had no prior history of smoking and denied any other symptoms. The physical examination was unremarkable and there was no known family history of malignancy. Esophagogastroduodenoscopy indicated an ulcerative lesion at the gastric greater curvature. Biopsy results showed ‘adenocarcinoma, tubular pattern, Intestinal type by Lauren, moderately differentiated (grade 2).’ The patient had chronic anemia, and CEA and CA 19-9 were normal.

**Figure 1:**
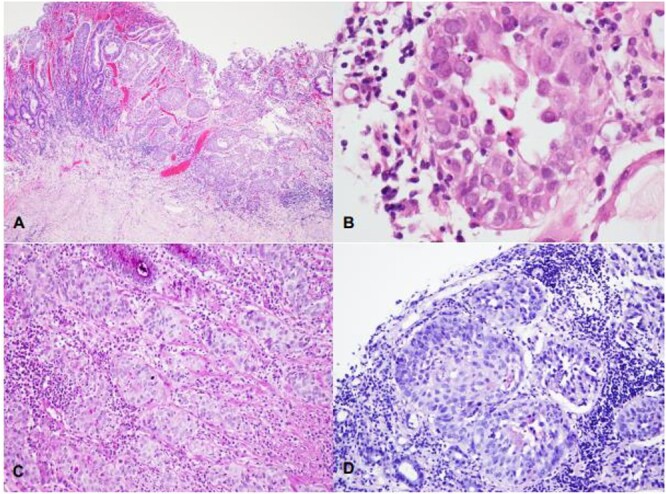
(A–D) Infiltration of the gastric mucosa by a solid neoplasm, organized in nests and composed by large, eosinophilic cells with moderate to marked nuclear pleomorphism (A—HE, 40X); foci of dyskeratosis may be seen (B—HE, 400X). Histochemical stains for mucin show a lack of staining in viable squamous cells (C—PAS, 100X; D—Mucicarmim).

**Figure 2:**
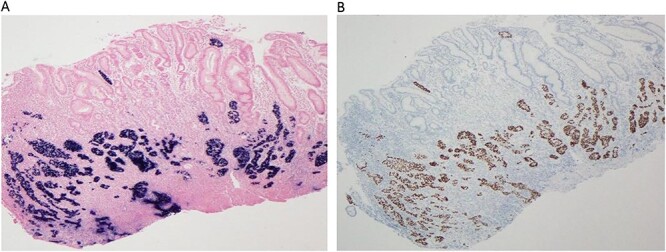
(A–B) Immunohistochemical study reveal tumor cells positive for P40 (A, 40X) and diffuse positivity for EBER *in situ* hybridization (B, 40X).

Abdominal, pelvic, and chest contrast-enhanced computed tomography (CT) revealed an extensive neoformative process involving the fundus, cardia, gastric lesser curvature, gastric fundus and gastric greater curvature with locoregional adenopathies in the lesser curvature and high lomboaortic. No metastases were detected. Full-body FDG PET/CT scan revealed gastric cancer (SUV 7.1), celiac adenopathy (SUV 4.5) and high left lomboaortic adenopathy (SUV 4.1). Staging laparoscopy showed no visible metastasis, and cytology was negative. Clinical staging was cT1N2M0. After a multidisciplinary meeting, peri-operative chemotherapy with fluorouracil, leucovorin, oxaliplatin and docetaxel (FLOT) was decided. Post-chemotherapy FDG PET/CT scan revealed soft caption on celiac adenopathy and high left lomboaortic adenopathy (SUV 3). The patient underwent total gastrectomy with Roux-en-Y reconstruction and D2 lymphadenectomy, along with caudal pancreatectomy and splenectomy due to suspicious involved lymph nodes on the pancreatic tail and splenic hilum. The postoperative period was uneventful, and she was discharged on the 10th postoperative day. Post-splenectomy vaccines were administered. The histopathological report confirmed PGSCC (p40+, EBER+, Ck7−) on the gastric corpus invading the serosa associated with Epstein–Barr virus (EBV) infection. The tumor exhibited infiltration of the gastric mucosa by a solid neoplasm, organized in nests, composed of large, eosinophilic cells with moderate to marked nuclear pleomorphism (Fig. 1A). Foci of dyskeratosis were also observed (Fig. 1B). Histochemical stains for mucin showed a lack of staining in viable squamous cells (Fig. 1C– D). Immunohistochemical study revealed tumor cells positive for P40 (Fig. 2A) and diffuse positivity for EBER in situ hybridization (Fig. 2B). Of the 26 resected lymph nodes, 18 were positive, and perineural and extramural venous invasion were present. Resection margins were free, and there was no response to neoadjuvant chemotherapy. The final TNM stage was ypT3 N3b M0 R0 (AJCC 8th edition). Adjuvant chemotherapy with 5-fluorouracil and cisplatin was continued. During follow-up, nodal and peritoneal recurrence was identified. The patient received several lines of treatment, including pembrolizumab, capecitabine plus irinotecan and paclitaxel, with disease progression in all cases. After 3 years of follow-up, she is still alive but is being followed up in palliative appointments.

## Discussion

PGSCC is an extremely rare entity, and our case, diagnosed in a female with no history of smoking, adds to its rarity. While most cases are diagnosed in men with a long history of smoking [1–5], our patient’s case becomes even more interesting. The etiopathology is still unknown, and several hypotheses have been proposed [2, 5, 6]. The histopathological report in our patient indicated PGSCC associated with EBV infection, a cause of 10% of all gastric cancers and also reported in PGSCC cases [5]. Similar to the majority of reported cases, our patient had no specific symptoms or signs, and the tumor was located in the upper third of the stomach, presenting as an ulcerative lesion [5–7]. Immunohistochemistry plays a crucial role in the diagnosis, as demonstrated in our patient, with positivity for CK5/6 and p40 [5–8]. No standard treatment strategy is defined by ESMO or NCCN [7, 9]. For non-metastatic disease, surgical resection R0 with D2 lymphadenectomy remains the main therapeutic approach and the only potential cure for localized disease [5–9]. Adjuvant chemotherapy regimens, including 5-fluorouracil-based or platin- and taxane-based regimens, have shown effectiveness [5–7, 9]. Limited data exist regarding neoadjuvant chemotherapy, but the FLOT regimen appears beneficial [8, 9]. Despite the initial histopathological diagnosis of adenocarcinoma, our patient underwent neoadjuvant chemotherapy with the FLOT regimen without a histopathological response [8, 9]. This raises questions about the true effectiveness of neoadjuvant therapy in treating SCC. The importance of reviewing results and the need for larger samples for an accurate diagnosis are reinforced by the incorrect initial histopathological diagnosis. Even after several lines of adjuvant treatment, our patient experienced disease progression. Most PGSCC cases are diagnosed at an advanced stage, highlighting the aggressiveness of this tumor [5–7].

## Conclusions

Early and accurate diagnosis, along with surgical treatment, are crucial for PGSCC. Our case raises questions about the relevance of performing neoadjuvant treatment in PGSCC. More data are needed to provide recommendations for the best treatment options for these patients.
